# Clinical experience in intracranial stenting of Wingspan stent system under local anesthesia

**DOI:** 10.3389/fneur.2024.1348779

**Published:** 2024-03-22

**Authors:** Mao-Shih Lin, Chih-Wei Huang, Yuang-Seng Tsuei

**Affiliations:** ^1^Department of Neurosurgery, Taichung Veterans General Hospital, Taichung, Taiwan; ^2^Institute of Medicine, Chung Shan Medical University, Taichung, Taiwan; ^3^Department of Post-Baccalaureate Medicine, College of Medicine, National Chung Hsing University, Taichung, Taiwan; ^4^Department of Neurosurgery, Tri-service General Hospital, National Defense Medical Center, Taipei, Taiwan

**Keywords:** intracranial stent, Wingspan, local anesthesia, angioplasty, intracranial atherosclerosis disease

## Abstract

**Objective:**

The use of endovascular treatments for symptomatic intracranial atherosclerosis disease (ICAD) remains contentious due to high periprocedural complications. Many centers resort to general anesthesia for airway protection and optimal periprocedural conditions; however, this approach lacks real-time monitoring of patients’ neurological status during procedures. In this study, we employed intracranial stenting with the Wingspan system under local anesthesia to address this challenge.

**Methods:**

We conducted a retrospective study of 45 consecutive ICAD patients who underwent intracranial stenting with the Wingspan system at our hospital from August 2013 to May 2021. These stenting procedures were performed under local anesthesia in a hybrid operation room. Neurological assessments were conducted during the procedure. The patients with periprocedural complications were analyzed for the risk factors.

**Results:**

The study included 45 ICAD patients (median age 62 years; 35 male and 10 female individuals). Among them, 30 patients had anterior circulation ICAD, and 15 had posterior circulation ICAD. The periprocedural complication rate was 8.9% (4/45), with an overall mortality rate of 2.2% (1/45). Notably, no procedure-related perforation complications were found, and all ischemic complications occurred in the perforating bearing artery, specifically in patients with stents placed in the middle cerebral artery or basilar artery, while no complications were observed in the non-perforating bearing artery of the internal carotid artery and vertebral artery (*p* = 0.04).

**Conclusion:**

Our study demonstrates the safety and efficacy of the Wingspan stent system when performed on selected patients under local anesthesia. This approach seems to reduce procedural-related morbidity and be a safe intervention. In addition, it is crucial for surgeons to be aware that patients with perforator-bearing artery stenosis may be at a higher risk of complications.

## 1 Introduction

Intracranial atherosclerotic disease (ICAD) represents a major cause of stroke, especially prevalent among Asian and Black populations, characterized by the atherosclerotic narrowing of intracranial arteries and resulting in reduced cerebral blood flow (1, 2). Despite the advancements in antithrombotic therapies, ICAD remains associated with a high annual stroke recurrence rate of 4–19%, underscoring the need for more effective treatments (3, 4). Neuroendovascular therapies, such as percutaneous transluminal angioplasty and stenting (PTAS), have emerged as promising alternatives. Specifically, the introduction of the self-expanding Wingspan stent system (Stryker, Kalamazoo, MI, United States) has marked a significant milestone, albeit with outcomes in clinical trials such as SAMMPRIS and VISSIT, which showed more complication rate in the stenting groups than in the medical treatment group, highlighting the risk of ICAD stenting (5, 6).

Subsequent studies, including the WEAVE trial, have suggested that procedural refinements and careful patient selection may significantly mitigate these risks, demonstrating a notably lower complication rate, which showed a low 2.6% periprocedural complication rate in 152 cases (7, 8). Nevertheless, the recent CASSISS trial emphasized the necessity of comparing these endovascular strategies against medical management, showing no significant benefit in stroke prevention but a lower complication rate with the Wingspan stenting from previous reports (9, 10).

A critical factor in the procedural risk of ICAD stenting is the use of general anesthesia, known to induce hemodynamic changes and preclude intraoperative monitoring of the patient’s neurological status (11, 12). Given the concerns regarding procedural-related complications and the impact of general anesthesia on perioperative outcomes, our institution has adopted a novel approach by performing all ICAD stenting procedures under local anesthesia. This study aims to evaluate the safety, efficacy, and complication rates of awake stenting using the Wingspan system, offering insights into its potential advantages over conventional methods.

## 2 Methods

### 2.1 Patient population

In this retrospective study, we examined the clinical data and electronic medical records of 45 patients who underwent awake endovascular intervention using the Wingspan stent system for ICAD at our institute between August 2013 and May 2021. All procedures were conducted by an experienced endovascular neurosurgeon, ensuring consistency and eliminating operator-related variables from the study’s scope.

#### 2.1.1 Inclusion criteria for Wingspan stenting

Patients eligible for Wingspan stenting met the following criteria:

- Symptomatic ICAD of at least 70% stenosis affecting the intracranial internal carotid artery, middle cerebral artery, intracranial vertebral artery, or basilar artery, as confirmed by digital subtraction angiography (DSA).
- History of transient ischemic attack or ischemic stroke with a modified Rankin scale (mRS) score of ≤2, specifically related to hypoperfusion territory evident on either magnetic resonance imaging (MRI) or perfusion computed tomography (CT) scan.
- Stenosis length not exceeding 15 mm, as assessed by DSA.
- Stroke occurrence more than 7 days prior to the procedure.
- Patient’s ability to cooperate with the awake stenting procedure.

#### 2.1.2 Exclusion criteria

Patients meeting any of the following criteria were excluded from the study:

- Presence of non-atherosclerotic stenosis.
- Concurrent intracranial lesions such as brain tumors or arterial malformations.
- Intolerance to antithrombotic therapy.

### 2.2 Stenting procedure

The stenting procedures were conducted under local anesthesia, omitting the use of sedatives or anesthetic agents to ensure the patient’s alertness and cooperation throughout the intervention. Patients were positioned supine on the procedure table, with their heads secured in place using medical tape to minimize head movement. To enhance patient comfort and provide a stable platform for the surgeon, a custom-made acrylic platform was employed. This platform facilitated the manipulation of the over-the-wire stent delivery system, especially during the exchange of microcatheters.

Prior to the procedure, a loading dose of 300 mg clopidogrel and 300 mg aspirin was administered. Systemic heparinization was achieved using 5,000 international units (IU) of heparin. An anesthesiologist and nurse anesthetists monitored the awake surgical procedure. The endovascular interventions took place in a state-of-the-art hybrid operating room equipped with an advanced angiography system (ARTIS Zeego, Siemens, Germany).

Under local anesthesia achieved through subcutaneous injection of 2% xylocaine at the puncture site, a 6-French sheath was inserted via the right femoral artery. Subsequently, a 6-French 070 Neuron catheter (Penumbra Inc., Alameda, CA, United States) was navigated to the distal part of the internal carotid artery (ICA) or vertebral artery (VA) to serve as the guide catheter. Using digital subtraction angiography (DSA) roadmap guidance, a Transcend 300-cm microwire (Boston Scientific, Natick, MA, United States) was advanced coaxially with a microcatheter (SL-10, Stryker, Kalamazoo, MI, United States) across the stenotic site with utmost care.

Following this, an Emerge Monorail Balloon Catheter (Boston Scientific, Natick, MA, United States) was carefully advanced to the stenotic lesions for balloon angioplasty. The balloon angioplasty was undersized, ensuring that the balloon diameter did not exceed 80% of the parent artery’s diameter. A staged angioplasty with slow inflation of the balloon gradually reaching 4 atmospheres (atm) of pressure over 30 s, followed by deflation and neurological function assessment, was performed. A second angioplasty was performed, reaching a nominal inflation pressure of 6 atm. Throughout this process, the surgeon and nurses vigilantly monitored the patient’s neurological condition. Any new-onset symptoms such as headache, slurred speech, limb weakness, or altered consciousness prompted an immediate pause in the procedure. Vital signs, including blood pressure, were closely monitored and maintained within the range of 100–150 mmHg. An anesthesiologist stood by in case a transition to general anesthesia became necessary. In the event of patient movement causing displacement of the angiography roadmap, the roadmap was repeated as needed.

Following balloon angioplasty, the Wingspan stent was advanced to the pre-stenotic site and carefully positioned, ensuring precision by confirming the anchoring point through repeat DSA roadmap guidance. Stent deployment was then executed meticulously. Subsequently, all the patients were transferred to the neuro-ICU or step-down unit, where strict blood pressure control (maintained between 100 and 120 mmHg) was enforced to prevent reperfusion hemorrhage. The procedure protocol is summarized in Table 1.

**Table 1:** Procedure protocol for Wingspan stenting under local anesthesia.

Pre-procedure preparation
Schedule the procedure more than 7 days of experiencing an acute event. Confirm patient cooperation pre-procedure and provide thorough education to ensure understanding and alleviate anxiety. Premedication with a loading dose of 300 mg clopidogrel and 300 mg aspirin.
Procedure Setup
Determine the necessity of Foley catheter insertion based on patient tolerance. Monitor blood pressure and heart rate frequently at 3–5-min intervals, maintaining systolic blood pressure within the range of 100–150 mmHg. Provide oxygen supplementation via nasal cannula. Employ head fixation. Systemic heparinization was achieved using 5,000 IU of heparin.
Anesthesia and access
Perform groin puncture under local anesthesia of xylocaine, avoiding intravenous fentanyl to minimize consciousness depression. The anesthesiologist was on standby.
Imaging
Utilize roadmap imaging frequently to ensure precise manipulation of microwires or microcatheters, particularly when the catheter passes stenotic areas.
Staged angioplasty
Perform angioplasty using an undersized balloon, approximately 80% of the original vessel diameter. First goal: gradually inflate the balloon, reaching 4 atm over 30 s, followed by deflation and neurological function assessment. In subsequent inflations, reach a nominal 6 atm over 30 s, followed by deflation and neurological function reassessment. Consider premature termination if a new neurological deficit arises or if the patient becomes uncooperative.
Stent deployment
After 3 min of clinical observation, advance the Wingspan stent to the lesion site for deployment. Avoid post-dilation.
Post-procedure care
Conduct post-operative 2D/3D DSA imaging. Achieve wound closure using appropriate devices. Sent the patient to the ICU or step-down unit with strict blood pressure control.

### 2.3 Clinical outcome assessment

In this study, demographic information, intraoperative findings, and periprocedural complications of the patients were meticulously extracted from the medical records. Periprocedural stroke and transient ischemic attack (TIA) were defined as new-onset neurological deficits occurring within 30 days of the procedure. These events were evaluated by neurologists. Periprocedural complications were categorized as follows:

Procedure-related complication: morbidity resulting specifically from the perforation of the vessel by use of microwires or microcatheters; hemorrhagic stroke: characterized by bleeding in the brain resulting in neurological symptoms; ischemic infarction event: refers to neurological deficits lasting longer than 24 h due to a lack of blood flow to specific brain regions; transient ischemic attack (TIA): denotes a temporary neurological event resolved within 24 h.

### 2.4 Statistical analysis

All statistical analyses were conducted using Statistical Package for the Social Sciences (SPSS) version 22.0 (IBM Corp., Armonk, NY, United States). To determine statistical significance, the Mann–Whitney U-test was employed for continuous variables, while the chi-square test was used for categorical variables. A *p* value less than 0.05 was considered statistically significant, indicating meaningful differences in the analyzed parameters.

## 3 Results

### 3.1 Basic characteristics

A total of 45 patients underwent successful Wingspan stenting procedures under local anesthesia; all stent deployments were completed without the need for conversion to general anesthesia, indicating the efficacy of this approach. Among these patients, a total of 49 stents were successfully deployed [17 in the vertebral–basilar artery (VBA) system and 32 in the anterior circulation]. The study group consisted of 35 male patients (77.8%) and 15 female patients (22.2%). Notably, there was a significant difference in the ratio of anterior circulation to posterior circulation stenosis stenting, with a 2:1 ratio. Male patients exhibited a higher prevalence of posterior circulation stenting (66.7 vs. 100.0%, *p* = 0.019). A detailed summary of the baseline characteristics and the analysis of patients in the complication and non-complication groups is presented in Tables 2, 3.

**Table 2:** Patient characteristics and clinical presentations.

Characteristics	Overall		Without complications		With complications		*p* value
Number of patients	45		41		4		
Age, years	62.8	(11.7)^**^	65	(56.0–70.0)^#^	68	(62.5–74.3)^#^	0.358
Sex							1
Male	35	(77.8%)	32	(78.0%)	3	(75.0%)	
Female	10	(22.2%)	9	(22.0%)	1	(25.0%)	
Hypertension	30	(66.7%)	26	(63.4%)	4	(100.0%)	0.285
Diabetes mellitus	18	(40.0%)	16	(39.0%)	2	(50.0%)	1
Hyperlipidemia	13	(28.9%)	11	(26.8%)	2	(50.0%)	0.567
History of coronary artery disease	9	(20.0%)	6	(14.6%)	3	(75.0%)	0.021^*^
Chronic kidney failure	1	(2.2%)	1	(2.4%)	0	(0.0%)	1
Presentation symptoms before stenting							0.059
Transient ischemic attack	12	(26.7%)	12	(29.3%)	0	(0.0%)	
Minor stroke	27	(60.0%)	25	(61.0%)	2	(50.0%)	
Major stroke	6	(13.3%)	4	(9.8%)	2	(50.0%)	
Lesion location							0.591
Anterior circulation	30	(66.7%)	2	(50.0%)	2	(50.0%)	
Posterior circulation	15	(33.3%)	13	(31.7%)	2	(50.0%)	

Chi-square test or Mann–Whitney U-test. ^*^p < 0.05; ^**^Standard deviation; ^#^Median (IQR).

**Table 3:** Patients presenting symptoms and lesion locations.

Characteristics	Overall		Without complications		With complications		*p* value
Presentation symptoms before stenting							0.059
Transient ischemic attack	12	(26.7%)	12	(29.3%)	0	(0.0%)	
Minor stroke	27	(60.0%)	25	(78.0%)	2	(50.0%)	
Major stroke	6	(13.3%)	4	(22.0%)	2	(50.0%)	
Lesion location							
Anterior circulation	30	(66.7%)	2	(39.0%)	2	(50.0%)	0.591
Posterior circulation	15	(33.3%)	13	(26.8%)	2	(50.0%)	0.591
Perforator bearing artery	21	(46.7%)	17	(14.6%)	4	(100.0%)	0.04^*^
Non-perforator bearing artery	24	(53.3%)	24	(2.4%)	0	(0.0%)	0.04^*^

The Chi-square test or Mann–Whitney U-test. ^*^p < 0.05.

### 3.2 Periprocedural complications

Periprocedural complications were noted in four patients: two patients (4.4%) with anterior circulation ICAD and two patients (4.4%) with posterior circulation ICAD. Specifically, one patient experienced a minor stroke during the procedure while under local anesthesia. Two others encountered delayed ischemic events. Notably, a case of reperfusion hemorrhage in the middle cerebral artery (MCA) territory following Wingspan stenting led to a fatality on the first day post-operation, contributing to an overall mortality rate of 2.2% (one out of 45 patients). Further information about these cases is detailed in Table 4.

**Table 4:** Patients with complications.

Patient number	Age	Sex	Stenosis location	Presenting symptoms before stent	Intraprocedural findings	Stent size	Postprocedural complication	Present symptoms	Brain imaging	Outcome
1	64	Male	Basilar artery	Acute right side pontine infarction	Severe stenosis at VBJ	Wingspan 3.5x15mm	TIA	Left side weakness	No new stroke	Stable
2	72	Male	Left M1-2	Acute left basal ganglia infarction, left frontal watershed infarction	Only one left MCA branch on exam	Wingspan 3.5x20mm	Hemorrhage stroke	conscious change	Massive intracranial hemorrhage	Death at POD 7
3	62	Female	Right M1	Recurrent TIA	Irregular stenosis at distal M1 segment right MCA	Wingspan 2.5x20mm	Infarction	Left side weakness	Right putaminal stroke	Hemiparesis
4	75	Male	Left VA & BA	Previous left basal ganglia infarction	Tandem lesion at left VA and lower third of BA	VA: Express SD 3.5 mm × 15 mm; BA: Wingspan 4.0 mm × 20mm	Infarction	Blurred vision, right cranial nerve III palsy	Multiple infarction: left occipital, left paramedian midbrain and pons	Stable

BA, Basilar artery; MCA, Middle cerebral artery; POD, Post-operative day; TIA, Transient ischemic attack; VA, Vertebral artery.

Three patients experienced ischemic strokes following the stenting procedure. Two of these cases were categorized as perforator strokes, and one presented with multiple cerebral and midbrain embolic infarcts. It is worth noting that all patients (4/4) who experienced complications, such as stroke or intracranial hemorrhage, had undergone stenting at the MCA/basilar artery (BA) territories, which are known to be “perforator-bearing” arteries. In contrast, none of the patients in this study underwent stenting at the internal carotid artery (ICA) or vertebral artery (VA) territories. Importantly, throughout our series, there were no incidents of procedure-related complications, such as wire perforation or artery dissection, underscoring the technical feasibility and safety of this study.

## 4 Discussion

The primary focus of this study was to explore the feasibility of performing elective intracranial stenting procedures using the awake PTAS technique with the Wingspan system. The Wingspan stent system, a self-expanding stent, designed for use with the Gateway angioplasty balloon, has been scrutinized for its role in cerebral artery revascularization and stroke prophylaxis. However, it is noteworthy that there is a lack of randomized trials investigating the impact of different anesthesia techniques on clinical outcomes and complications in intracranial stenting procedures. Despite this gap in research, many medical institutions continue to administer intracranial stents under general anesthesia, with only a handful of studies indicating the feasibility of using the Wingspan system or other coronary stents under local anesthesia (13–16).

The SAMMPRIS study protocol has stipulated the necessity of performing the stenting procedure under general anesthesia to ensure patient immobility during the operation (6, 11, 17). However, there are concerns regarding the potential complications associated with general anesthesia. Utilizing local anesthesia for Wingspan intracranial stenting offers a clear advantage, primarily in avoiding the need for endotracheal intubation and minimizing hemodynamic fluctuations during induction. These factors are crucial, especially in patients with severe intracranial stenosis, as they have been linked to perioperative strokes (6, 11). By using local anesthesia, confounding variables originating from general anesthesia can be effectively eliminated, providing a safer alternative for patients undergoing intracranial stenting procedures.

From the surgeon’s perspective, dealing with severe stenosis in ICAD patients presents challenges due to poor image quality in the DSA roadmap caused by reduced blood flow. Manipulating the microcatheter to straighten parent vessels can inadvertently lead to unintended microwire entry into the wrong vessels, resulting in iatrogenic perforation (14). Drawing from our experience, we found that frequent updates of the DSA roadmap offer the most accurate wire delivery, crucial in preventing catastrophic complications. Additionally, real-time interaction with patients and monitoring neuralgic conditions provide invaluable feedback during critical steps such as angioplasty or passing the guidewire through the stenotic site, especially in challenging cases. Notably, our study did not observe intraoperative subarachnoid hemorrhage from wire perforation.

However, the use of local anesthesia in intracranial stenting raises concerns among surgeons. It may pose technical difficulties due to the risk of patient movement during surgery, potentially lowering the technical success rate. Frequent roadmap updates might be necessary to compensate for patient movements. In our study, we employed a custom-made head holder, similar to other research groups, to immobilize the patient during procedures (15). Additionally, we utilized a custom-made “acrylic platform” positioned over the patient’s trunk, providing additional support for the 300-cm wire and the entire stenting system. This setup guards against accidental or abrupt movements of the distal tip, preventing subarachnoid hemorrhage when changing catheters (18). By securely holding the wire on the acrylic platform with the assistant’s assistance, surgeons can confidently transition to the Wingspan stent system, alleviating concerns about inadequate wire support or wire-tip movement. Moreover, this approach reduces the discomfort experienced by conscious patients during the procedure.

In our study, we achieved a 100% technical success rate in awake stenting, an acceptable periprocedural complication rate of 8.9%, a mortality rate of 2.2%, and, notably, no incidents of procedure-related complications such as wire perforation or vessel rupture due to angioplasty. Compared to previously reported data on intracranial stents performed under conscious sedation or local anesthesia, Chamczuk et al. (15) demonstrated a 12.1% periprocedural complication rate and a 3.2% mortality rate, while Abou-Chebl et al. (13) reported a 14.6% periprocedural complication rate and a 2.5% mortality rate. Yu et al. (14) found a major stroke or death rate of 4.2% and a total periprocedural complications rate of 9.5%. Furthermore, Jiang et al. (16) reported using the Wingspan stent for vertebrobasilar stenosis, demonstrating the ability to timely identify neurological conditions in patients, with low complication rates in the posterior circulation at 7%, representing a significant improvement compared to historic controls. Among these studies, the procedure-related complication rate, specifically wire perforation ranged from 1.5 to 6.2%. Our study exhibited promising results. Therefore, the overall results of intracranial stenting under local anesthesia suggest effectiveness and safety.

The endovascular recanalization procedure for extracranial internal carotid artery stenosis under conscious sedation or local anesthesia has become a widely accepted practice. We consider the application of the Wingspan stent system under local anesthesia as a natural extension of this approach. For experienced surgeons, performing delicate stenting procedures under local anesthesia can potentially result in safer and more successful outcomes for patients. Utilizing the patient’s consciousness, surgeons can actively assess brain perfusion status, a crucial factor in such intricate procedures. Evidence from a retrospective study on carotid artery stenting (CAS) revealed that patients under general anesthesia had 2.3 times higher odds of in-hospital mortality and twice the odds of extended hospital length of stay when compared to those under local anesthesia (19). Many surgeons prefer employing local anesthesia for CAS procedures as it allows for superior neurological assessment (19, 20). Similarly, in the case of Wingspan stenting, which involves high-risk manipulation of intracranial vessels, local anesthesia offers surgeons an advantage to evaluate the patient’s condition during the procedure. Furthermore, there is some degree of hypoperfusion and unintentional hyperventilation in the neuro-endovascular procedure in general anesthesia, which may lead to cerebral ischemia and induce poor outcomes (11, 12).

Complications arising from intracranial stenting using the Wingspan system often stem from perforator territories (4, 10, 18, 21). Several mechanisms have been postulated to explain these complications. First, according to a simulated biomechanical model of the Wingspan stent, structural deformation of the perforator orifice can occur after stent deployment, potentially leading to perforator stroke (22). Second, the theory of the “snow-plowing effect” suggests that stent expansion displaces the atheroma plaque occluding perforators, increasing the risk of perforator infarction (16, 23). Finally, the placement of the Wingspan stent system can induce the straightening of the tortuous vessels, which can cause kinking and overstretching of perforators, impairing blood flow, and resulting in ischemia (16). Given these hypotheses, it is challenging to prevent all perforator strokes, even with an experienced surgeon. Therefore, it becomes crucial to focus on reducing the risk of perforator infarction, especially in “perforator-bearing” arteries such as MCA and BA, to enhance the clinical outcomes of ICAD procedures. In our study involving patients under local anesthesia, we observed that perforator strokes occurred in only one patient in a perforator-bearing artery, which was a lower incidence than in other studies. Under local anesthesia, surgeons can potentially mitigate the risk by avoiding oversize angioplasty, closely monitoring the patients’ neurological conditions of patients, and reducing distal vessel ischemia. This vigilant approach may contribute significantly to minimizing complications related to perforator infarction.

### 4.1 Limitations

While the study highlighted the advantage of awake intracranial stenting using the Wingspan stent system, there are several limitations to consider. First, the study’s retrospective nature means that it lacks a direct comparison between patients undergoing the procedure under local anesthesia and those under general anesthesia. This absence of a comparative analysis limits the ability to draw definitive conclusions regarding the superiority of one anesthesia method over the other. Second, there might be a selection bias influenced by the surgeon’s experience, leading to variations in the decision to perform intracranial stent procedures. Finally, the study’s sample size is relatively small, which raises concerns about the generalizability of the findings. Further research involving larger and more diverse patient groups is essential.

## 5 Conclusion

Local anesthesia in intracranial stenting procedures with the Wingspan system offers distinct advantages including real-time monitoring of the patient’s neurological status and a potential reduction in the risk of perforator stroke. In our series, it also indicated no procedure-related complications, such as wire perforation. While this study sheds light on the potential benefits of using local anesthesia and awake stenting using the Wingspan system, surgeons should remain cautious among patients with perforator-bearing artery stenosis, as they may have a higher risk of complications.

## Data availability statement

The raw data supporting the conclusions of this article will be made available by the authors, without undue reservation.

## Ethics statement

The studies involving humans were approved by Taichung Veteran General Hospital. The studies were conducted in accordance with the local legislation and institutional requirements. The participants provided their written informed consent to participate in this study.

## Author contributions

M-SL: Writing – original draft. C-WH: Writing – review & editing. Y-ST: Supervision, Validation, Writing – original draft, Writing – review & editing.
